# Regulation of LPS-Induced Inflammatory Responses in Bovine Mammary Epithelial Cells via TLR4-Mediated NF-κB and MAPK Signaling Pathways by Lactoferrin

**DOI:** 10.3390/life15010069

**Published:** 2025-01-09

**Authors:** Kai Zhang, Ruizhen Zhang, Yuanyuan Zhang, Min Zhang, Hong Su, Feifei Zhao, Daqing Wang, Guifang Cao, Yong Zhang

**Affiliations:** 1 College of Veterinary Medicine, Inner Mongolia Agricultural University, Hohhot 010010, China; zhangkai040423@163.com (K.Z.); 13238430585@163.com (R.Z.); zyyworkaccount@163.com (Y.Z.); zhangmin5400@126.com (M.Z.); hongsu1995@126.com (H.S.); imauzff@126.com (F.Z.); wangdaqing050789@126.com (D.W.); 2 Animal Embryo and Developmental Engineering Key Laboratory of Higher Education, Institutions of Inner Mongolia Autonomous Region, Hohhot 010011, China; 3 Inner Mongolia Autonomous Region Key Laboratory of Basic Veterinary Medicine, Hohhot 010011, China; 4 College of Life Sciences, Inner Mongolia University, Hohhot 010011, China

**Keywords:** bovine mammary epithelial cells, lactoferrin, lipopolysaccharide, signal path

## Abstract

Lactoferrin (LF), a member of the transferrin family, is widely present in mammalian milk and other secretions, exhibiting anti-inflammatory, antibacterial, and anti-infective properties. Although the biological functions of LF have been extensively studied, there are few reports on its effects and molecular mechanisms concerning bovine mastitis caused by bacterial infection. This study used bovine mammary epithelial cells (BMECs) cultured in vitro as the research model. An inflammatory injury model was established by stimulating BMECs with LPS to investigate whether LF at different concentrations (10, 50, 100, and 200 μg·mL^−1^) could inhibit the inflammatory response before and after the onset of inflammation. The expression of inflammatory cytokines IL-1β, IL-6, IL-8, and TNF-α at both the gene and protein levels was detected using RT-qPCR and ELISA. Western blotting was employed to evaluate the phosphorylation levels in the inflammatory signaling pathways MAPK/P38/ERK and NF-κB/P65, while RT-qPCR was used to examine the impact on TLR4 receptor gene expression. The results display that pretreatment with LF prior to LPS-induced inflammation in BMECs reduced the expression of inflammatory cytokines IL-1β, IL-6, IL-8, and TNF-α at both the gene and protein levels (*p* < 0.05). LF also inhibited the phosphorylation of NF-κB/P65 and MAPK/P38/ERK signaling pathways and downregulated TLR4 receptor gene expression (*p* < 0.05). However, when LF was added after the onset of LPS-induced inflammation, inflammatory cytokine expression and phosphorylation levels in the NF-κB/P65 and MAPK/P38/ERK pathways remained elevated, along with high expression of the TLR4 receptor gene (*p* < 0.05). These findings show that LF can antagonize LPS-induced inflammatory responses in BMECs and reduce cytokine expression, exhibiting anti-inflammatory effects when administered before inflammation. Conversely, when LF is added post-inflammation, it appears to enhance cytokine expression, potentially promoting the recruitment of more cells or factors to resolve inflammation rapidly. Both effects are mediated through the TLR4 receptor and the NF-κB/P65 and MAPK/P38/ERK signaling pathways.

## 1. Introduction

Bovine mastitis is a common and highly prevalent disease that poses a serious threat to dairy cattle health and causes significant economic losses to the global livestock industry. Annually, approximately $35 billion is spent worldwide on mastitis treatment [1]. Halasa et al. reported that cows with acute mastitis incur an annual cost of $367 per cow, while those with subclinical mastitis incur an average annual cost of $130 per cow [2]. Thus, preventing bovine mastitis is of paramount importance. The causes of bovine mastitis are multifactorial, including pathogenic microorganisms, environmental hygiene factors, host-specific factors, and nutritional deficiencies, with pathogenic microbial infections being the primary cause [3]. Pathogenic microorganisms typically enter the mammary gland via the teat duct, where they colonize the epithelial barrier, triggering inflammation [4]. Rudenko et al. isolated 486 pathogenic microorganisms from the mammary glands of 103 cows with mastitis, identifying *Escherichia coli* and *Staphylococcus aureus* as the primary bacterial culprits [5].

Lipopolysaccharide (LPS) is a major component of the cell wall of Gram-negative bacteria, such as *E. coli*, and a key virulence factor in Gram-negative bacterial infections in animals. As such, LPS is widely recognized as an important inducer of systemic inflammation [6]. When Gram-negative bacteria invade the body, they release LPS, which binds to CD14 and MD2 and is subsequently recognized by Toll-like receptor 4 (TLR4), thereby activating TLR4-mediated signaling pathways. This activation triggers the nuclear factor-κB (NF-κB) and mitogen-activated protein kinase (MAPK) pathways, leading to the secretion of pro-inflammatory cytokines, including IL-1β, IL-6, and TNF-α, as well as chemokines like IL-8. These molecules recruit and activate immune cells, initiating the body’s immune response [7]. TLRs are critical signal-transducing membrane proteins in innate immunity and inflammation, serving as the first line of defense against pathogenic microorganisms. Their activation can result in the destruction of invading pathogens [8].

Lactoferrin (LF) is a cationic metal-binding glycoprotein primarily expressed by epithelial cells and found in various bodily secretions, such as semen, pancreatic fluids, tears, saliva, uterine secretions, and milk. The highest concentration of LF is found in colostrum [9]. LF is also expressed by innate immune cells, such as neutrophils, and plasma LF is considered to be derived from neutrophils. Under normal conditions, its levels are low, but they can increase severalfold during bacterial infections and in the presence of tumors [10,11]. Many of LF’s immunomodulatory properties are linked to its ability to bind pathogen-associated molecular patterns (PAMPs), which are recognized by specific receptors, such as TLRs, on innate immune cells. Studies have reported that LF can bind to LPS and unmethylated CpG motifs [12,13]. LPS, as a prototypical PAMP, exhibits high affinity for the lactoferrin C-lobe (LFc) domain due to its lipid A structure [14]. This binding not only involves immune cells, such as monocytes/macrophages, neutrophils, and lymphocytes, but also endothelial cells, all of which recognize LPS through specific receptors like TLR4 to block LPS signaling [15]. Furthermore, LF has been shown to have a high affinity for soluble CD14, which acts as a co-receptor for TLR4 in the recognition of LPS [16]. These interactions demonstrate that LF suppresses the activation of cells at inflammatory sites induced by LPS. This suppression results in reduced expression of pro-inflammatory cytokines, such as TNF-α, IL-6, and IL-1 in immune cells, and decreased levels of adhesion molecules and IL-8 in endothelial cells. The most direct evidence for LF’s role in host defense comes from studies on LF deficiency in humans and animal models where LF supplementation was shown to provide protection against infections, allergic inflammation, and cancer [17]. However, there have been few reports on LF’s role in treating bovine mastitis, and LF’s mechanism of action in this context remains unclear. Based on the above analysis, we assume that LF plays a therapeutic role in preventing bacterial mastitis in dairy cows.

Therefore, this study aimed to investigate the anti-inflammatory effects of LF on LPS-stimulated BMECs as a model of inflammation. Different concentrations of LF (10, 50, 100, and 200 μg·mL**^−^**^1^) were assessed for their ability to suppress inflammatory responses in BMECs, both before and after the onset of LPS-induced inflammation. Using techniques such as ELISA, RT-qPCR, and Western blot, the study analyzed the expression of inflammatory cytokines (IL-1β, IL-6, IL-8, and TNF-α) at the gene and protein levels, the activation of NF-κB and MAPK signaling pathways, and the expression of the TLR4 receptor gene. This research aims to provide a basis for the clinical application of LF in the prevention of bacterial bovine mastitis, in order to provide the theoretical basis for immune enhancers and feed additives for dairy cows.

## 2. Materials and Methods

### 2.1. Reagents, Chemicals, and Antibodies

The following materials were used: DMEM/F12 medium, PBS, and fetal bovine serum (FBS) (Hyclon, Logen, UT, USA); LPS (Sigma Aldrich, St. Louis, MO, USA); holo-lactoferrin from bovine milk was 97% pure (product number: L9507, certificate of analysis: SLCH5940, Sigma, MO, USA); bovine TNF-α, IL-8, IL-6, and IL-1β ELISA kits (Qidibio, Wuhan, China); SDS-PAGE loading buffer (Takara, Shiga, Japan); M-PER mammalian protein extraction reagent, pre-stained protein ladders, Halt Protease Inhibitor, Starting Block T20 (TBS) Blocking Buffer (Thermo Fisher Scientific, Waltham, MA, USA); Pierce BCA Protein Assay Kit (Beyotime, Shanghai, China); SDS-PAGE gel electrophoresis kit (Solarbio, Beijing, China); Axy Prep Multi-source Total mRNA Miniprep Kit (Axygen Scientific, Union City, NJ, USA); Primer Script RT Master Mix (Vazyme, Nanjin, China); epidermal growth factor, bovine insulin, transferritin, hydrocortisone, progesterone, recombinant bovine placental prolactin, penicillin-streptomycin (MCE, Monmouth Junction, NJ, USA), ERK, phospho-ERK, p38, phospho-p38, p65, phospho-p65, β-actin (Cell Signaling Technology, Beverly, Danvers, MA, USA). The primers used were designed and synthesized by Sangon Biotech (Shanghai, China).

### 2.2. Source of Bovine in Mammary Epithelial Cells

The cells used in this experiment were all BMECs cultured in our laboratory. All the cells used in this experiment were fifth-generation cells. The formula of the whole culture medium is epidermal growth factor (10 μg·mL^−1^), bovine insulin (5 μg·mL^−1^), transferritin (5 mg·mL^−1^), hydrocortisone (5 mg·mL^−1^), progesterone (1 μg·mL^−1^), recombinant bovine placental prolactin (10 ng·mL^−1^), penicillin-streptomycin (1%), and fetal bovine serum (10%).

### 2.3. Experimental Infection

**Figure 1:**
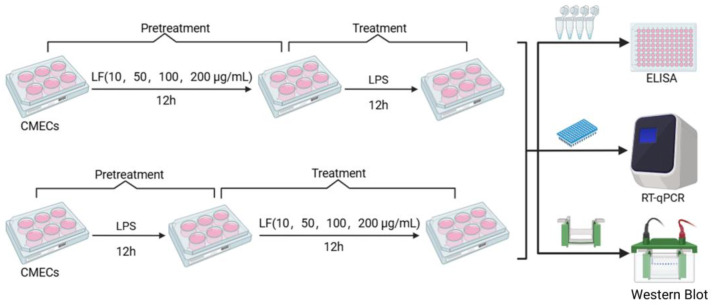
Experimental design for the effect of LF treatment or pretreatment in bovine mammary epithelial cells.

The medium was discarded after treating cells with LF (10, 50, 100, and 200 μg·mL^−1^) for 12 h, and then the cells were treated with LPS for 12 h, or the medium was discarded after treating cells with LPS for 12 h and then the cells were treated with LF (10, 50, 100, and 200 μg·mL^−1^) for 12 h (Figure 1).

### 2.4. Enzyme-Linked Immunosorbent Assay (ELISA)

According to the instructions provided by different cytokine ELISA kits, different cytokines are detected separately, and then the concentration of each cytokine sample is calculated by drawing the corresponding standard curve. Three biological replicates were performed for each experiment.

### 2.5. Cell Viability Assay

The activity of BMECs was determined by the CCK-8 method. BMECs were placed in 96-well plates with a density of 1 × 10^4^ cells per well and incubated in a 5% CO_2_ cell incubator at 37 °C for 24 h. BMECs were incubated with designated concentrations of LF (10, 50, 100, and 200 μg·mL^−1^) for 4, 8, 12, and 24 h. The BMECs were then incubated with 10 μL CCK-8 solution and 100 μL DMEM/F12 medium incubated in the dark for 4 h. The absorbance was measured at 450 nm by an enzyme-labeled instrument.

### 2.6. Western Blot Analysis

The cells were lysed with M-PER and the total protein was extracted. Its concentration is determined and denatured. At 80 V, the proteins on each channel were separated using SDS-PAGE electrophoresis (12%). The protein was transferred to polyvinylidene fluoride membranes at 25 V for 30 min. At room temperature, the membranes were blocked with TBST containing 3% BSA for 4 h. The different primary antibodies were diluted to the appropriate concentration and incubated at 4 °C for 14 h. After incubation, the membranes were washed 5 times with TBST for 5 min. The secondary antibody was incubated at room temperature for 1 h. Then, TBST was used to wash the membranes three times for 10 min each time. After this, they were exposed to electrochemiluminescence film and Western blot detection tools. Image J (V1.8.0.112) was used, and band densities were measured.

### 2.7. Real-Time PCR Analysis

The total mRNA of cells was extracted and reverse-transcribed. The reaction conditions were as follows, for 40 cycles: 50 °C, 2 min; 95 °C, 10 min; 95 °C, 15 s; 60 °C, 60 s. The annealing temperature was 58 °C. The primers used are listed in Table 1. The results were calculated using the 2^−∆∆Ct^ calculation method.

### 2.8. Statistical Analysis

All data had been analyzed with the use of GraphPad Prism 8 and are expressed as the mean ± standard deviation (SD). Statistical significance was evaluated by one-way analysis of variance (ANOVA) followed by Tukey’s multiple comparisons test or two-way ANOVA (Bonferroni’s post-test), as appropriate. Differences with *p* values ≤ 0.05 were considered statistically significant.

## 3. Results

### 3.1. Effect of LF on the Activity of Bovine Mammary Epithelial Cells 

**Figure 2:**
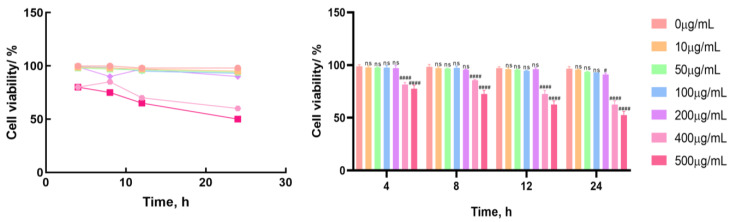
Effect of LF on the activity of bovine mammary epithelial cells. LF with concentrations of 0, 10, 50, 100, 200, 400, and 500 μg·mL^−1^ were used to treat BMECs in all groups individually. Cell viability was measured using the CCK-8 method. Results are presented as mean ± standard deviation of three independent experimental data, with Bonferroni post hoc testing performed for two-factor analysis of variance (ns *p* > 0.05, # *p* < 0.05 and #### *p* < 0.0001 compared to the control group).

The potential cytotoxicity of LF to BMECs at different time points (4, 8, 12, and 24 h) and concentrations (0, 10, 50, 100, 200, 400, and 500 μg·mL^−1^) was evaluated by the CCK-8 method. Within a 12 h treatment period of LF at a concentration range of 0–200 μg·mL^−1^, BMECs presented no significant changes in their viability, indicating that LF exhibited no potential cytotoxic effect on BMECs within this period and this concentration range (Figure 2). Therefore, this concentration range can be used to measure the influence of LF on BMECs within a treatment period of 12 h.

### 3.2. Pretreatment with LF Reduces LPS-Induced Inflammatory Responses in BMECs

**Figure 3:**
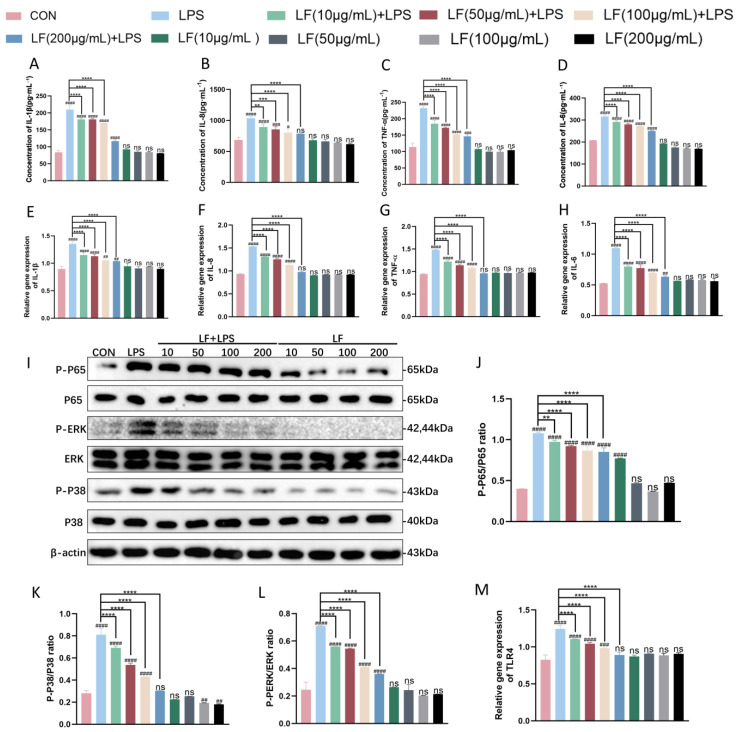
Pretreatment with LF reduces LPS-induced inflammatory responses in BMECs. BMECs were pretreated with various concentrations of LF (10, 50, 100, and 200 μg·mL^−1^) for 12 h before stimulation with LPS for another 12 h. (**A**–**D**) Concentrations of IL-1β, IL-8, TNF-α, and IL-6 in BMEC culture supernatants were measured using ELISA. (**E**–**H**) Expression levels of IL-1β, IL-8, TNF-α, and IL-6 were detected using RT-qPCR. (**M**) TLR4 receptor gene expression was measured using RT-qPCR. (**I**–**L**) Phosphorylation levels in NF-κB/P65 and MAPK/P38/ERK signaling pathways were detected by Western blot. The results are presented as the mean ± standard deviation (SD) of three independent experiments. One-way ANOVA with Bonferroni post hoc tests was used for statistical analysis. (ns *p* > 0.05, # *p* < 0.05, ## *p* < 0.01, ### *p* < 0.001, and #### *p* < 0.0001 compared to control group. ns *p* > 0.05, ** *p* < 0.01, *** *p* < 0.001, and **** *p* < 0.0001 compared to LPS-only group).

To evaluate whether LF can mitigate the inflammatory response induced by LPS in BMECs, different concentrations of LF (10, 50, 100, and 200 μg·mL^−1^) were added to BMECs 12 h prior to LPS stimulation. Subsequently, the expression levels of inflammatory cytokines (IL-1β, IL-8, TNF-α, and IL-6) at both gene and protein levels, phosphorylation levels in inflammatory signaling pathways NF-κB/P65 and MAPK/P38/ERK, and the expression of the TLR4 receptor gene were measured. The results showed that compared to the LPS-only group, the LF+LPS group (i.e., the group pretreated with LF before LPS stimulation) exhibited significantly reduced expression of inflammatory cytokines (IL-1β, IL-8, TNF-α, and IL-6) at both gene and protein levels, as well as significantly reduced phosphorylation levels in NF-κB/P65 and MAPK/P38/ERK signaling pathways and TLR4 receptor gene expression (Figure 3). Additionally, the reduction became more pronounced as the concentration of LF increased, demonstrating a dose–response relationship. The most significant reduction was observed at an LF concentration of 200 μg·mL^−1^ (**** *p <* 0.0001). These findings indicate that pretreatment with LF effectively reduces the inflammatory response induced by LPS in BMECs.

### 3.3. Post-Treatment with LF Promotes LPS-Induced Inflammatory Responses in BMECs

**Figure 4:**
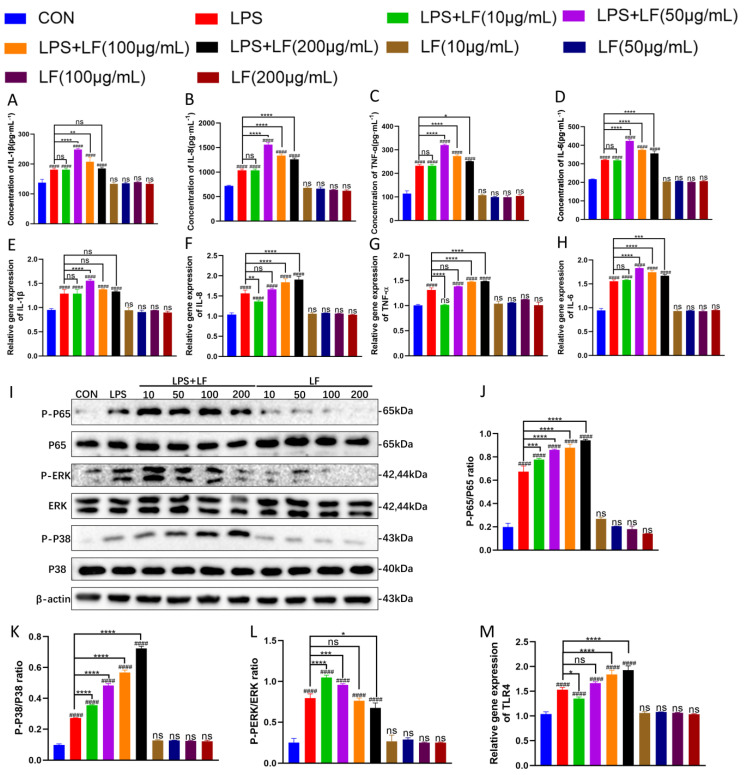
Post-treatment with LF promotes LPS-induced inflammatory responses in BMECs. BMECs were first stimulated with LPS for 12 h, followed by treatment with different concentrations of LF (10, 50, 100, and 200 μg·mL**^−1^**) for another 12 h. (**A**–**D**) Concentrations of IL-1β, IL-8, TNF-α, and IL-6 in the BMEC culture supernatants were measured by ELISA. (**E**–**H**) Gene expression levels of IL-1β, IL-8, TNF-α, and IL-6 were detected by RT-qPCR. (**M**) TLR4 receptor gene expression was measured by RT-qPCR. (**I**–**L**) Phosphorylation levels in NF-κB/P65 and MAPK/P38/ERK signaling pathways were assessed by Western blot. The results are expressed as the mean ± standard deviation (SD) of three independent experiments. Statistical analysis was performed using one-way ANOVA with Bonferroni post hoc tests. (ns *p* > 0.05, and #### *p* < 0.0001 compared to control group. ns *p* > 0.05, * *p* < 0.05, ** *p* < 0.01, *** *p* < 0.001, and **** *p* < 0.0001 compared to LPS-only group).

To assess whether LF affects the inflammatory response in BMECs after LPS stimulation, different concentrations of LF (10, 50, 100, and 200 μg·mL^−1^) were applied to BMECs 12 h after LPS stimulation for another 12 h. The expression of inflammatory cytokines (IL-1β, IL-8, TNF-α, and IL-6) at both gene and protein levels, phosphorylation levels in inflammatory signaling pathways NF-κB/P65 and MAPK/P38/ERK, and the expression of the TLR4 receptor gene were evaluated. The results showed that, compared to the LPS-only group, the expression levels of IL-1β and IL-6 at both the gene and protein levels (Figure 4A,E,D,H) and the protein levels of IL-8 and TNF-α (Figure 4B,C) in the LPS+LF group (i.e., the group treated with LF after LPS stimulation) first increased and then decreased. At LF concentrations of 50, 100, and 200 μg·mL^−1^, the protein levels of IL-8 and TNF-α and the gene and protein levels of IL-6 were significantly increased, exceeding those of the LPS-only group. Additionally, the protein level of IL-1β exceeded that of the LPS-only group at LF concentrations of 50 and 100 μg·mL^−1^, while its gene expression level exceeded that of the LPS-only group at an LF concentration of 50 μg·mL^−1^. It was observed that the gene and protein levels of IL-1β and IL-6 and the protein expression levels of IL-8 and TNF-α in the LPS+LF group were highest at the LF concentration of 50 μg·mL^−1^ (**** *p <* 0.0001). However, the gene expression levels of IL-8 and TNF-α (Figure 4F,G) increased with higher concentrations of LF added to the LPS-only group. When LF with concentrations of 100 and 200 μg·mL^−1^ were added to the LPS-only group, the expression levels in the LPS+LF group exceeded those of the LPS-only group, with the highest expression observed at 200 μg·mL^−1^ (**** *p <* 0.0001). In the NF-κB and MAPK inflammatory signaling pathways, it was observed that, compared to the LPS-only group, the phosphorylation levels of P-P65 and P-P38 increased with higher LF concentrations in the LPS+LF group, exceeding those of the LPS-only group and peaking at 200 μg·mL^−1^ (Figure 4J,K) (**** *p <* 0.0001). However, the phosphorylation level of P-PERK exceeded that of the LPS-only group at LF concentrations of 10 and 50 μg·mL^−1^ (Figure 4L). The expression level of the TLR4 receptor gene increased with higher LF concentrations in the LPS+LF group, exceeding that of the LPS-only group at LF concentrations of 100 and 200 μg·mL^−1^ and peaking at 200 μg·mL^−1^ (Figure 4M) (**** *p <* 0.0001).

These findings demonstrate that, compared to the LPS-only group, the application of different concentrations of LF after LPS stimulation resulted in varying degrees of increased expression of inflammatory cytokines (IL-1β, IL-8, TNF-α, and IL-6) and elevated phosphorylation levels in NF-κB/P65 and MAPK/P38/ERK pathways, as well as TLR4 receptor gene expression. However, not all of these changes exhibited a dose–response relationship. The results indicate that the addition of LF at different concentrations following LPS stimulation can promote the inflammatory response in LPS-stimulated BMECs.

## 4. Discussion

Mastitis is a disease that severely affects dairy cow health, reduces milk yield, and compromises milk quality. Various pathogenic factors can cause mastitis, with bacterial infections being the primary cause among environmental factors. Common bacteria such as *S. aureus* and *E. coli* can rapidly multiply after entering mammary tissues through the teat canal, leading to mastitis [18]. Therefore, preventing and managing bacterial mastitis in dairy cows is a significant public health challenge. LPS, a major component of Gram-negative bacteria, elicits an inflammatory response similar to that caused by intact *E. coli*, promoting the secretion of inflammatory factors and leading to inflammation [19]. LF, an important protein found in milk and widely present in biological secretions, has demonstrated various physiological activities, including immunomodulatory and anti-inflammatory effects [20]. However, the specific functions and mechanisms of LF in LPS-induced BMECs and its role in defending against mastitis remain unclear, warranting further investigation.

Numerous studies have shown the multiple biological activities of LF and that it is well tolerated in humans, with minimal adverse reactions. As a result, LF is used in many countries as a nutritional supplement for anti-tumor effects, immune enhancement, and memory improvement. Evidence of LF’s role in host defense is derived from human LF deficiency and animal models supplemented with LF, indicating LF’s protective effects against infections, allergic inflammation, and cancers [17]. The concentrations of LF used vary across different experimental designs. For example, Liliana et al. used LF concentrations of 50–500 μg/mL in THP-1 cell lines [21], while Moussa et al. used a concentration of 500 μg/mL in studies on *S. aureus* invasion of mammary epithelial cells [22]. Based on these findings, we used the CCK-8 assay to examine the effects of various concentrations of LF (0, 10, 50, 100, 200, 400, and 500 μg/mL) on BMECs’ viability. The results showed no significant changes in BMECs’ viability after 12 h of treatment with LF concentrations ranging from 0 to 200 μg/mL, indicating no potential cytotoxicity of LF within this time and concentration range (Figure 2). This concentration range was thus deemed appropriate for assessing LF’s effects on BMECs over 12 h. The slight differences in results compared to those of Liliana et al. and Moussa et al. may be attributed to variations in cell types and experimental conditions, which are considered normal deviations.

During mastitis, mammary tissues exhibit elevated levels of inflammatory factors such as TNF-α, IL-6, IL-8, and IL-1β. These cytokines and chemokines play critical roles in the pathophysiology caused by bacterial infections [19]. LPS, a major component of the outer membrane of Gram-negative bacteria, has been used to construct mastitis models by adding it to cultured BMECs to mimic the inflammatory processes induced by bacterial infection [19]. Therefore, this experiment also employs this model. TLR4, a transmembrane protein and upstream receptor of NF-κB, specifically recognizes PAMPs such as LPS [23]. Studies have reported that LPS can induce inflammatory responses through the TLR4–NF-κB pathway. As a key nuclear transcription factor, NF-κB plays a critical role in regulating the expression of inflammatory cytokines [24]. Previous research has demonstrated that LF can exert its anti-inflammatory effects by inhibiting the binding of LPS to TLR4 [25]. For instance, LF has been shown to alleviate endotoxin-induced bone damage by inhibiting TLR4-mediated immune responses [26]. Additionally, LF can regulate NF-κB structure and function, thereby modulating NF-κB activity to suppress inflammation. Detection of P-P38, P-PERK, and P-P65 in mice with LPS-induced acute lung injury treated with *Codonopsis pilosula* polysaccharides (CPPs) revealed that CPPs alleviate lung tissue damage in mice by inhibiting the MAPK and NF-κB pathways, reducing the phosphorylation levels of P-P38, P-PERK, and P-P65, and thereby improving lung function in mice [27]. In clinical trials conducted by Paesano’s team, treatment with bovine LF and ferrous sulfate for pregnant women with hereditary thrombosis-related iron deficiency and iron deficiency anemia showed that serum IL-6 levels continuously decreased in those treated with bovine LF, with no adverse reactions [28]. Moussa et al. found that LF reduced LPS-induced cytokine production in monocytes via the NF-κB signaling pathway [21], while Diarra et al. reported that bovine LF inhibited the invasion of *S. aureus* into BMECs [22].

Based on these studies, we used *E. coli* LPS to stimulate BMECs and construct an inflammatory model to explore whether LF could attenuate LPS-induced inflammatory responses in BMECs through TLR4-mediated NF-κB and MAPK signaling pathways. The results showed that compared to the control group, the expression levels of inflammatory cytokines IL-1β, TNF-α, IL-8, and IL-6 were significantly elevated at both the gene and protein levels in the LPS-only group. These findings successfully replicated the in vivo inflammatory processes in vitro, confirming the successful establishment of the inflammation model (Figure 3A–H). When different concentrations of LF were added prior to LPS stimulation, the TLR4 receptor gene expression level was significantly reduced compared to the LPS-only group (Figure 3M). Additionally, the expression levels of inflammatory cytokines IL-1β, TNF-α, IL-8, and IL-6 were significantly decreased at both the gene and protein levels (Figure 3A–H). The phosphorylation levels of proteins in TLR4-mediated NF-κB and MAPK signaling pathways were also significantly reduced, and these reductions were positively correlated with the concentration of LF (Figure 3I–L). These results align with previous findings, demonstrating that LF mitigates LPS-induced inflammation in BMECs by modulating the production of inflammatory cytokines through the TLR4-mediated NF-κB and MAPK pathways, thereby antagonizing LPS-induced inflammatory responses.

To further explore whether LF can regulate the inflammatory response of BMECs via the TLR4 receptor-mediated NF-κB and MAPK signaling pathways after inflammation has already occurred in BMECs, we first induced inflammation in BMECs with LPS and then added LF. We observed changes in TLR4 receptor gene expression, the gene and protein expression levels of inflammatory cytokines IL-1β, IL-6, IL-8, and TNF-α, as well as the phosphorylation levels of proteins in the TLR4 receptor-mediated NF-κB and MAPK signaling pathways. The results showed that after LPS stimulation of BMECs, the addition of various concentrations of LF led to a significant increase in TLR4 receptor gene expression, gene and protein expression levels of inflammatory cytokines IL-1β, IL-6, IL-8, and TNF-α, and phosphorylation levels of proteins in the NF-κB and MAPK pathways (Figure 4). This suggests that after LPS stimulation of BMECs, adding LF promotes the occurrence of LPS-induced BMEC inflammation. We hypothesize that once inflammation occurs in the body, it requires the participation of a large number of cells and complexes that control the inflammation. These cells are attracted to the site of inflammation by the release of high levels of inflammatory factors such as TNF-α. TNF-α is an important pro-inflammatory cytokine that appears early during the acute phase of inflammation, activating the secretion of other inflammatory cytokines such as IL-1 and various adhesion factors. IL-1 activation is the initial step of the body’s specific immune response and marks the beginning of the fight against infection. IL-6 accelerates macrophage maturation and enhances the immune activity of B cells [29]. IL-8, a chemotactic factor, mediates early tissue damage and activates neutrophils and T cells. IL-1β is also a key factor involved in inflammation [30]. Therefore, it seems that only when these factors are further increased can the body better resist infection and eliminate inflammation. LF may promote the production of cytokines in BMECs, thereby accelerating the resolution of inflammation.

Some studies have found that LF participates in both innate and adaptive immunity. It helps the host resist microbial invasion and protects the host from the destructive effects of inflammation. However, in different disease states and stages of the inflammatory response, LF exhibits different effects in different species, displaying both anti-inflammatory and pro-inflammatory properties. The regulation of NF-κB by LF is mostly inhibitory, but it can also be stimulatory under certain conditions [31]. This view supports our hypothesis. Other studies have found that the effect of LF on upregulating or downregulating NF-κB expression may be related to LF concentration. Low concentrations of bovine LF block NF-κB activation and reduce IL-8 secretion, showing an anti-inflammatory effect, while high concentrations of LF exhibit pro-inflammatory effects by upregulating NF-κB expression [32]. This view differs from our results, as we found that when LF is added before inflammation occurs, it helps prevent LPS-induced BMEC inflammation, with increasing anti-inflammatory effects as the LF concentration rises. However, when LF is added after LPS-induced BMEC inflammation has already occurred, the overall effect on BMECs is pro-inflammatory, with no clear evidence of low concentrations exerting anti-inflammatory effects and high concentrations showing pro-inflammatory effects.

## 5. Conclusions

In summary, before LPS-induced BMEC inflammation occurs, LF can counteract LPS-induced inflammation by reducing the expression of inflammatory factors, showing an anti-inflammatory effect. After LPS-induced inflammation has occurred, LF can promote inflammation by increasing the expression of inflammatory factors to attract more cells or factors involved in the inflammatory response, thereby accelerating inflammation resolution. Both effects are mediated through the TLR4 receptor-mediated NF-κB/P65 and MAPK/P38/ERK signaling pathways. These results assess the anti-inflammatory effects of LF at the cellular level, providing new potential therapeutic methods for bacterial mastitis in dairy cows and a theoretical basis for the clinical application of LF.

## Figures and Tables

**Table 1:** Primer sequences for RT-qPCR.

Gene Name	Sequences (5′-3′)	Accession Number
β-actin	F: 5′-CCAAGGCCAACCGTGAGAAGAT-3′ R: 5′-CCACGTTCCGTGAGGATCTTCA-3′	NM_173979.3
TNF-α	F: 5′-CAACGGTGTGAAGCTGGAAGAC-3′ R: 5′-TGAAGAGGACCTGTGAGTAGATGAG-3′	NM_173966.3
Il-1β	F: 5′-ATGAAGAGCTGCATCCAACACCTG-3′ R: 5′-ACCGACACCACCTGCCTGAAG-3′	NM_174093.1
IL-8	F: 5′-GCTGGCTGTTGCTCTCTTGG-3′ R: 5′-GGGTGGAAAGGTGTGGAATGTG-3′	NM_173925.2
IL-6	F: 5′-ATGATGAGTGTGAAAGCAGCAAGG-3′ R: 5′-TGATACTCCAGAAGACCAGCAGTG-3	NM_173923.2

## Data Availability

Data are contained within the article.
